# Prokinetic Activity of Mulberry Fruit, *Morus alba* L.

**DOI:** 10.3390/nu15081889

**Published:** 2023-04-14

**Authors:** Tae Sik Sung, Seung-Bum Ryoo, Chang-Hyun Lee, Seon-Min Choi, Joo-Won Nam, Hyun-Bok Kim, Ji young Lee, Jung-Dae Lim, Kyu Joo Park, Hyun-Tai Lee

**Affiliations:** 1 Department of Surgery, Seoul National University College of Medicine, Seoul 03080, Republic of Korea; slover1209@gmail.com (T.S.S.); sbryoomd@gmail.com (S.-B.R.); kjparkmd@snu.ac.kr (K.J.P.); 2 Biopharmaceutical Engineering Major, Division of Applied Bioengineering, College of Engineering, Dong-Eui University, Busan 47340, Republic of Korea; leechang5389@naver.com (C.-H.L.); tjsals0808@naver.com (S.-M.C.); 3 College of Pharmacy, Yeungnam University, Gyeongsan 38541, Republic of Korea; jwnam@yu.ac.kr; 4 National Institute of Agricultural Sciences, Rural Development Administration, Wanju 55365, Republic of Korea; hyunbok@korea.kr; 5 Department of Ophthalmology and Visual Science, Daejeon St. Mary’s Hospital, College of Medicine, The Catholic University of Korea, Seoul 06591, Republic of Korea; ram1020@naver.com; 6 Department of Herbal Medicine Resource, Kangwon National University, Samcheok 25949, Republic of Korea; ijdae@kangwon.ac.kr; 7 Core-Facility Center for Tissue Regeneration, Dong-Eui University, Busan 47340, Republic of Korea

**Keywords:** intestinal transit, *Morus alba*, mulberry fruit, myogenic contraction, neurogenic contraction, prokinetic, smooth muscle

## Abstract

The fruit of *Morus alba* L. (MAF) has been consumed as a food worldwide. MAF has also been widely used in traditional medicine for thousands of years in East Asia, and its diverse bioactivities have been reported in numerous publications. However, no prokinetic activity has been reported for MAF or its components. In the present study, therefore, we investigated the effects of MAF on gastrointestinal motor function by measuring the intestinal transit rate (ITR) of Evans blue in mice in vivo. The ITR values accelerated by MAF were significantly higher than those accelerated by cisapride or metoclopramide, suggesting that MAF has potential as a new prokinetic agent to replace cisapride and metoclopramide. We also investigated the effects of MAF on myogenic and neurogenic contractions in human intestinal smooth muscles by measuring spontaneous contractions of smooth muscle strips, smooth muscle contractions induced by neural stimulation, and migrating motor complexes from intestinal segments in the human ileum and sigmoid colon in situ. MAF increased both myogenic and neurogenic contractions to enhance ileal and colonic motility in the human intestine. Taken together, these results indicate that MAF enhanced intestinal motility by increasing both myogenic and neurogenic contractions, thereby accelerating the ITR.

## 1. Introduction

*Morus alba* L., known as white mulberry or silkworm mulberry, is a deciduous tree widely cultivated in many regions of the world, including East Asia [1,2,3]. Due to its chemical composition and pharmacological activities, various parts of *M. alba*, such as the leaves, root barks, branches, and fruits, have been used in traditional oriental medicine for thousands of years [4,5]. Among them, the fruit of *M. alba* (abbreviated as “MAF” in this paper) is commonly eaten fresh, dried, or processed into wine, fruit juice, jam, and canned food due to its delicious taste, pleasing color, low-calorie content, and high nutrient content [1,6]. In East Asia, MAF has also been used for a long time as a traditional medicine to alleviate hyperglycemia, hypertension, fever, and sore throat; protect the liver and kidney from damage; improve eyesight; strengthen the joints; facilitate the discharge of urine; moisten dryness; and treat other disorders [1,7,8,9]. Modern researchers have validated many potential health benefits of MAF and/or its bioactive ingredients, such as anthocyanins, flavonoids, polyphenols, alkaloids, and polysaccharides, which showed a variety of bioactive functions in vitro and in vivo, including antioxidant [10,11], antiatherosclerosis [12,13], immunomodulatory [14,15], anticancer [16,17], antihyperglycemic [18,19], hypolipidemic [20,21], and neuroprotective [22] activities. Although the effects of MAF and/or its components on the gastrointestinal (GI) tract have been reported mainly for their anti-inflammatory activity [23,24], little is known about their effects on GI motility. Only a few studies have been reported for the effects of different parts of *M. alba* (i.e., leaves and root barks) on GI motor functions [25,26], but prokinetic (or GI motility-related) activity has not been reported for MAF or its components.

The term “prokinetic” refers to a drug or its action that enhances GI motility by increasing the frequency or strength of contractions. Unlike cancers or cardiovascular diseases, GI motility disorders are not immediately fatal to health. However, these diseases causing daily discomfort often severely reduce the quality of life in the current era of well-being. Although significant advances have been made in recent basic and clinical research in the field of GI motility, causal treatment of motility disorders remains largely unresolved. Moreover, the most effective prokinetic agents were withdrawn from the market or restricted from use due to their significant side effects. Cisapride, the best-selling prokinetic in the late 1900s, was withdrawn from the market in 2000 due to its fatal cardiac side effects [27]. Similarly, in 2014, the use of metoclopramide and domperidone, other popular prokinetic agents comparable to cisapride, were restricted for functional GI tract disorders [28].

The discovery of new prokinetic agents is very important as no potent alternative to cisapride has yet been developed. In addition, a new drug should have a favorable safety profile as well as a well-proven efficacy. Because MAF has been eaten as a food for thousands of years, its safety has already been secured. In the present study, therefore, our aims were to investigate the effects of MAF on GI motor function. To do so, first, the intestinal transit rate (ITR) of Evans blue dye was measured in mice in vivo. The effect of MAF on the ITR was examined and compared with that of cisapride or metoclopramide. Spontaneous contractions of smooth muscle strips, smooth muscle contractions induced by neural stimulation, and the migrating motor complexes (MMCs) from intestinal segments were then recorded in the human ileum and sigmoid colon in situ, and the effects of MAF on myogenic and neurogenic smooth muscle contractions in human intestine were examined.

## 2. Materials and Methods

### 2.1. Drugs and Reagents

Catechin, cisapride, Folin–Ciocalteu reagent, metoclopramide hydrochloride, Nω-nitro-L-arginine (L-NNA), and quercetin were purchased from Sigma-Aldrich Co. (St. Louis, MO, USA). Evans blue and methylcellulose (MC) were purchased from Shanghai Aladdin Bio-Chem Technology Co., Ltd. (Shanghai, China). MRS 2500 tetraammonium salt was purchased from Tocris Bioscience (Ellisville, MO, USA). The other reagents used in this study were of analytical grade or better and were used without further purification.

### 2.2. Preparation and Quantitative Analysis of MAF

Fresh fruits of mulberry tree, *Morus alba*, were purchased from a farmhouse in Wanju, Korea, and lyophilized at −50 °C for 24 h using a freeze-drier (FDT-8612; Operon Co., Ltd., Gimpo, Republic of Korea) to obtain MAF in the form of dry powder. To assure consistent quality of MAF, total polyphenols and flavonoids in MAF were determined by the Folin–Ciocalteu method and the aluminum chloride calorimetric assay [29,30], respectively, with minor modifications. Catechin was used as a standard material for the content analysis of total polyphenols in MAF. A total of 0.5 g of MAF was soaked in 30 mL of 80% ethanol with shaking for 20 min and the suspension was ultrasonicated at 70 °C for 1 h and centrifuged (Centrifuge 5804R, Eppendorf AG, Hamburg, Germany) at 4000 rpm for 15 min. A total of 1 mL of the filtered supernatant (MAF-E80) or a standard solution of catechin was mixed with 1 mL of 1 N Folin–Ciocalteu reagent and 8 mL of distilled water followed by addition of 1 mL of 15% Na_2_CO_3_ solution. The mixture was then allowed to stand for 2 h at room temperature and the absorbance was measured at 760 nm using a UV-1800 Spectrophotometer (Shimadzu, Kyoto, Japan). The amount of total polyphenols was calculated and expressed as catechin equivalent (i.e., mg of cathechin/100 g of MAF). For the content analysis of total flavonoids in MAF, quercetin was used as a standard material. A total of 0.5 mL of MAF-E80 (or a standard solution of quercetin) was added to 2 mL of distilled water followed by addition of 0.15 mL of 5% NaNO_2_, 0.15 mL of 10% AlCl_3_·6H_2_O, and 1 mL of 1 M NaOH solutions. The mixture was then stirred for 5 min and allowed to stand for 15 min at room temperature, and the absorbance was measured against prepared reagent blank at 510 nm. The amount of total flavonoids was expressed as quercetin equivalent (i.e., mg of quercetin/100 g of MAF).

### 2.3. Animals

Five-week-old male ICR mice were obtained from Samtako Bio Korea Co., Ltd. (Osan, Republic of Korea). All animals were housed individually in clear plastic cages at Core-Facility Center for Tissue Regeneration, Dong-eui University (Busan, Republic of Korea). The animals were maintained under conditions of controlled temperature (22 ± 2 °C), humidity (55 ± 5%), and illumination (light on 7 a.m. to 7 p.m.). All animal experiments conducted in this study were performed in accordance with guidelines established by the Institutional Animal Care and Use Committee of Dong-eui University and approved by the committee (approval number: R2022-002). All mice were acclimated to the housing conditions with ad libitum access to a commercial diet and tap water for at least two weeks. The animals were then deprived of food with free access to tap water for 20 h before experimentation. At the onset of the experiment, mice weighed 25–35 g. All animal experiments were conducted between 10 a.m. and 5 p.m.

### 2.4. Measurement of ITR of Evans Blue

The effect of MAF on intestinal propulsion was assessed by measuring the intestinal transit of an Evans blue solution (5%, *w*/*v*, in DW) in mice in vivo, according to a previous method [31] with minor modifications. Cisapride and metoclopramide were selected as drugs for positive control groups. Twelve mice were tested for each dose of a test drug. An aqueous suspension of 0.5% MC was used as a vehicle. A 0.5% MC suspension containing each test drug (i.e., MAF, cisapride, or metoclopramide) was administered orally at each dose to conscious mice through an orogastric tube at a volume of 10 mL/kg body weight, and 30 min later, 0.1 mL of the Evans blue solution was administered orally to the mice through an orogastric tube. The animals were then sacrificed at 30 min after the administration of the Evans blue solution, and the intestinal transit of the Evans blue during the 30 min period was determined by measuring the distance that Evans blue migrated in the intestine from the pylorus to the most distal point of the intestine. Intestinal transit was expressed as intestinal transit rate (ITR), the percentage of the distance traveled by Evans blue divided by the total length of the small intestine (i.e., from the pylorus to the ileal end).

### 2.5. Acquisition and Preparation of Human Intestinal Tissues

Human bowel specimens were obtained from patients who underwent non-obstructive bowel cancer surgery at Seoul National University Hospital (SNUH). Ileum and sigmoid colon specimens were obtained from right hemicolectomy and anterior resection, respectively [32]. This study was approved by the Institutional Review Board (IRB) of the Clinical Research Institute of the SNUH (IRB approval No. H-0603-071-170). The study protocol was performed in accordance with the guidelines and regulation of the SNUH IRB. Written informed consent was obtained from all patients prior to operations. After resection of the ileum and sigmoid colon, the specimens were immediately transferred to preoxygenated Krebs-Ringer bicarbonate (KRB) solution containing (in mM) 15.5 NaHCO_3_, 120.4 NaCl, 5.9 KCl, 11.5 glucose, 2.5 CaCl_2_, 1.2 NaH_2_PO_4_, and 1.2 MgCl_2_. This solution was sufficiently oxygenated with 97% O_2_ and 3% CO_2_ and adjusted to pH 7.3–7.4 as described previously [33,34].

### 2.6. Tension Recordings of Smooth Muscle Strips

Contractions of smooth muscle strips were recorded in human ileum and sigmoid colon in situ, according to a previous method [33] with minor modifications. After removing the mucosal and submucosal layer of ileum and sigmoid colon, muscle layer was cut into strips of 5–6 mm in length and 2–3 mm in width. The muscle strips were mounted to an isometric force transducer (Biopac Systems, Inc., Goleta, CA, USA) and suspended in 10 mL organ bath containing aerated (97% O_2_ and 3% CO_2_) and warmed (36.5 ± 0.5 °C) KRB solution. The muscle strips were stabilized for 60 min without a force and equilibrated for 60 min after stretching the strips to 1 mN, in the presence of L-NNA (100 μM) and MRS 2500 (1 μM) to eliminate inhibitory responses. Electrical field stimulation (EFS; 10 Hz, 10 s, 100 V, 0.3 ms pulse) was applied to evoke neural response using a Grass S88 stimulator (Grass instruments, Warwick, RI, USA).

### 2.7. Tension Recordings of Bowel Segments

MMCs from intestinal segments were recorded in human ileum and sigmoid colon in situ, according to a previous method [34] with minor modifications. Ileal or colonic specimens with intact mucosal and submucosal layer were cut longitudinally in segments of 5–6 cm in length and 2 cm in width. Tension of circular muscle in each segment was recorded at 3 sites (proximal, middle, and distal sites, 2 cm apart) via perpendicular traction using sutures placed at each site (see Figure S1). Sutured muscle was connected to an isometric force transducer (Biopac Systems, Inc., Goleta, CA, USA) using threaded micro serrefines (Fine Science Tools, Forster City, CA, USA). The segments were equilibrated for at least 2 h before experiments after stretching the segments to 10 mN. Aerated (97% O_2_ and 3% CO_2_) and warmed (36.5 ± 0.5 °C) KRB solution was perfused continuously into the tissue chamber.

### 2.8. Data Acquisition and Analysis for Contractility in Human Intestinal Smooth Muscles

The mechanical responses were recorded and digitized using Acknowledge software (Biopac Systems, Inc., Goleta, CA, USA). Data were analyzed offline using Clampfit (version 10.2. Molecular devices, San Jose, CA, USA). Area under the curve (AUC), amplitude, and frequency for 5 min were analyzed for spontaneous contractions. AUC and amplitude for 10 s were analyzed for EFS-induced contractions. AUC for 10 min was analyzed for ileal or colonic MMCs. The contractility before MAF administration was adopted as a control value, and the change in contractility after MAF administration was calculated as a relative value (i.e., % of control) for comparison [33,34].

### 2.9. Statistical Analysis

All data of each experimental group are expressed as the mean ± standard error of the mean (SEM). Statistical analysis was performed using Prism 9.0 (GraphPad, Boston, MA, USA). Data were evaluated by one-way analysis of variance, followed by a Dunnett’s post hoc test, if appropriate. Statistical significance was considered when the *p*-value was less than 0.05.

## 3. Results and Discussion

### 3.1. Contents of Total Polyphenols and Flavonoids in MAF

In order to assure the consistency of the quality of the MAF used in the present study, the contents of the total polyphenols and flavonoids were measured in the MAF. The amounts of the total polyphenols and flavonoids in 100 g of MAF were calculated to be 1016.70 ± 48.03 mg of catechin (*n* = 3) and 550.39 ± 13.97 mg of quercetin (*n* = 3), respectively, suggesting a fairly good consistency in the quality of MAF in terms of the contents of total polyphenols and flavonoids.

### 3.2. Effect of MAF on ITR in Mice

**Figure 1:**
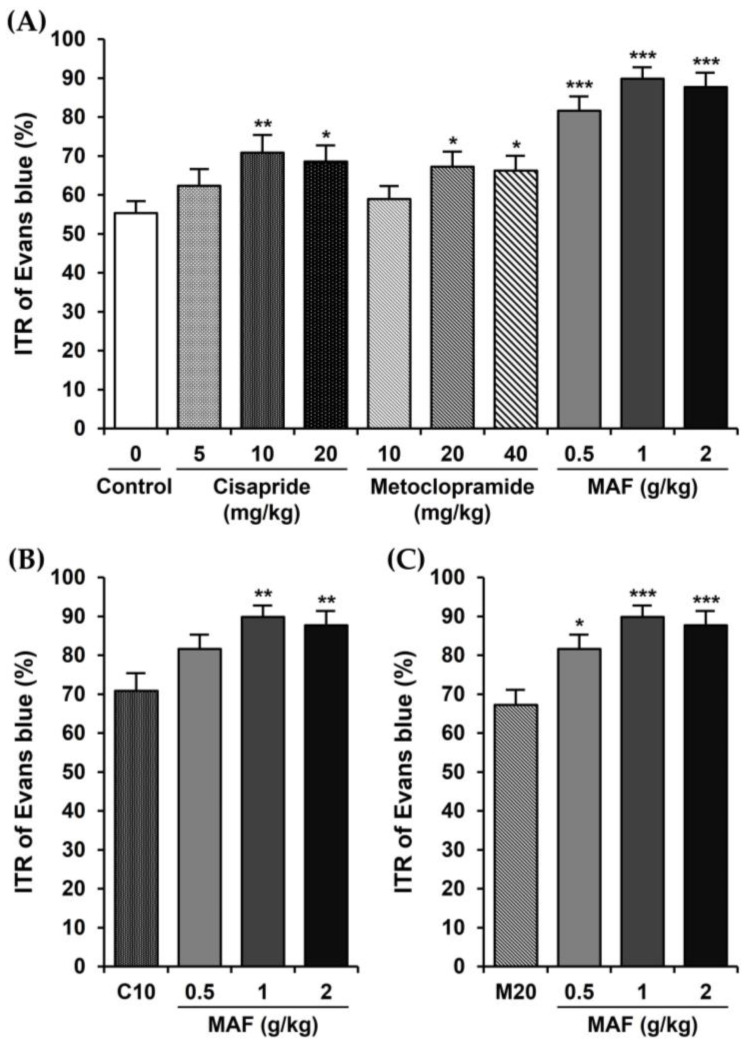
Effects of cisapride, metoclopramide, and MAF on ITR in mice in vivo. The ITR (%) of Evans blue during a 30 min period following oral administration of an Evans blue solution 30 min after pretreatment with each test drug at each dose in mice (*n* = 12 for each bar). Data were evaluated by one-way analysis of variance, followed by a Dunnett’s post hoc test, if appropriate. (**A**) Significant difference (* *p* < 0.05, ** *p* < 0.01, and *** *p* < 0.001) compared with control, (**B**) significant difference (** *p* < 0.01) compared with C10 (ITR in the cisapride-treated group at a dose of 10 mg/kg), (**C**) significant difference (* *p* < 0.05 and *** *p* < 0.001) compared with M20 (ITR in the metoclopramide-treated group at a dose of 20 mg/kg).

The ITRs (%) of the Evans blue during the 30 min period in mice are shown in Figure 1. The ITR for the mice with the treatment of only 0.5% MC as a vehicle (i.e., control group) was 55.33 ± 3.13%. The ITR was significantly accelerated when cisapride was administered orally at a dose of 10 or 20 mg/kg. The ITR value at a cisapride dose of 10 mg/kg (70.84 ± 4.60%, *p* < 0.01, compared to the control value) was higher than that of 20 mg/kg (68.55 ± 4.11%, *p* < 0.05) or 5 mg/kg (62.35 ± 4.34%, *p* > 0.05), suggesting that a maximally effective oral dose of cisapride might be around 10 mg/kg (Figure 1A). The ITR values in the metoclopramide-treated group also showed a similar pattern to those in the cisapride-treated group (i.e., 58.92 ± 3.33% (*p* > 0.05), 67.26 ± 3.89% (*p* < 0.05), and 66.21 ± 3.86% (*p* < 0.05), respectively, for 10, 20, and 40 mg/kg doses of metoclopramide). Thus, a maximally effective oral dose of metoclopramide might be around 20 mg/kg (Figure 1A). In comparison, the ITR values for the MAF at doses of 0.5, 1, and 2 g/kg were 81.58 ± 3.74, 89.81 ± 2.99, and 87.69 ± 3.66%, respectively. The ITR was increased significantly by the MAF at all doses (*p* < 0.001) compared to the control value (i.e., 55.33 ± 3.13%), and a maximally effective oral dose of MAF might be around 1 g/kg (Figure 1A). Moreover, in terms of the ITR, the efficacy of MAF was much higher than that of cisapride or metoclopramide with statistical significance (Figure 1B,C), implying that MAF has potential as a new prokinetic agent to replace cisapride and metoclopramide.

### 3.3. Effects of MAF on Spontaneous Contractions of Smooth Muscle Strips in Human Intestine

**Figure 2:**
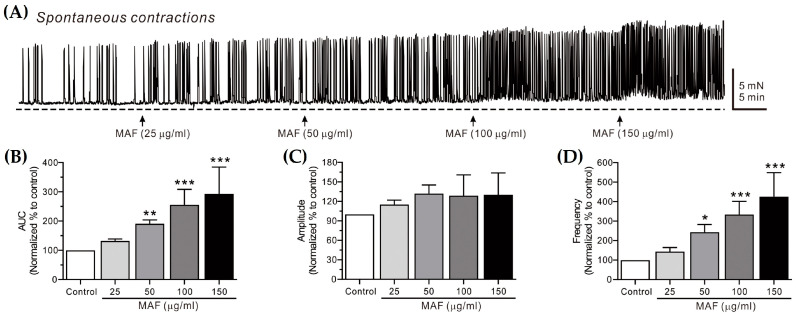
Effects of MAF on spontaneous contractions of smooth muscle strips in human ileum in situ. (**A**) Representative raw traces showing that MAF increased spontaneous contractions in a dose-dependent manner (25–150 μg/mL). Arrows indicate the points of MAF administration. Summarized graphs showing the effects of MAF on (**B**) AUC, (**C**) amplitude, and (**D**) frequency of spontaneous contractions. Significant difference (* *p* < 0.05, ** *p* < 0.01, and *** *p* < 0.001) compared with control.

**Figure 3:**
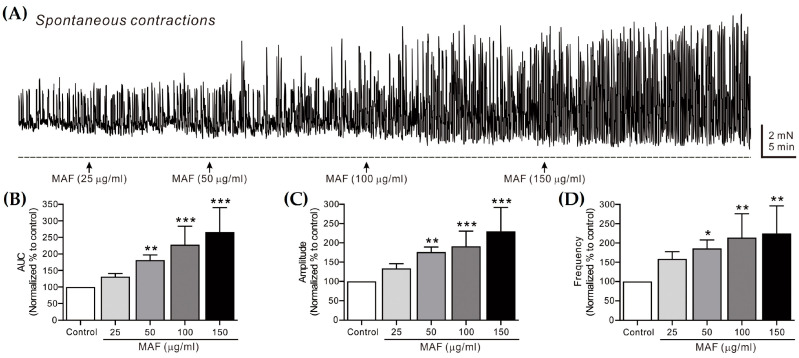
Effects of MAF on spontaneous contractions of smooth muscle strips in human sigmoid colon in situ. (**A**) Representative raw traces showing that MAF increased spontaneous contractions in a dose-dependent manner (25–150 μg/mL). Arrows indicate the points of MAF administration. Summarized graphs showing the effects of MAF on (**B**) AUC, (**C**) amplitude, and (**D**) frequency of spontaneous contractions. Significant difference (* *p* < 0.05, ** *p* < 0.01, and *** *p* < 0.001) compared with control.

The spontaneous contractions of smooth muscle strips were recorded in situ in the human ileum (Figure 2) and sigmoid colon (Figure 3) and their AUC, amplitude, and frequency for 5 min were analyzed (Table 1). The AUC and frequency for the ileal spontaneous contractions increased significantly after the administration of MAF in a dose-dependent manner, whereas the amplitude showed a slight increase with no statistical significance after the MAF administration at any dose, suggesting that the MAF-induced increase in the total contractility in the ileum was primarily due to an increase in the frequency rather than the amplitude of the spontaneous contractions. For the colonic spontaneous contractions, in comparison, the administration of MAF increased all three parameters (i.e., AUC, amplitude, and frequency) significantly in a dose-dependent manner, suggesting that the MAF-induced increase in the total contractility in the sigmoid colon was due to increases in both the amplitude and frequency of the spontaneous contractions.

Spontaneous contractions are caused by slow waves generated by the pacemaker interstitial cells of the Cajal (ICC) in the GI tract [35]. Because the spontaneous contractions occur without neural input in the smooth muscle layer, they are referred to as myogenic contractions [36]. In this study, the pattern by which MAF increased the myogenic contractions was somewhat different in the ileum and sigmoid colon, as described above. Nevertheless, MAF increased the myogenic contractions in both the ileum and sigmoid colon, implying that MAF directly affects smooth muscle cells or the ICC. Next, we investigated whether MAF affects neurogenic contractions that indirectly activate smooth muscle cells by stimulating enteric nerves.

### 3.4. Effects of MAF on EFS-Induced Contractions in Human Intestinal Smooth Muscles

**Figure 4:**
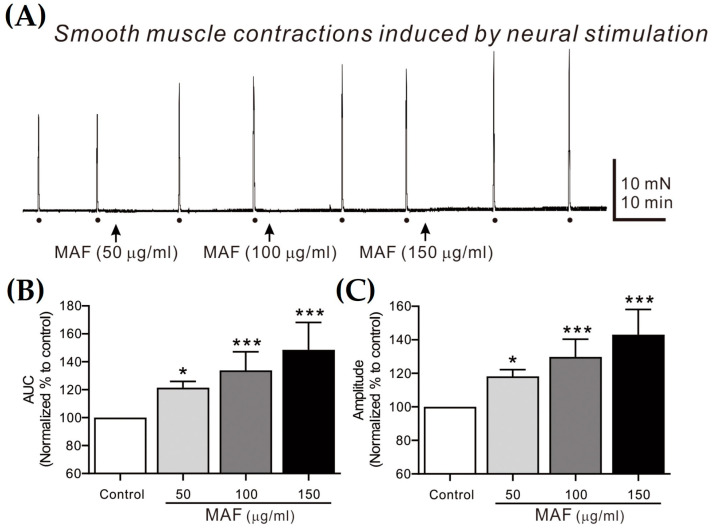
Effects of MAF on EFS-induced contractions of smooth muscle strips in human ileum in situ. (**A**) Representative raw traces showing that MAF increased electrically evoked contractions by neural stimulation in a dose-dependent manner (50–150 μg/mL). Dots and arrows indicate the points of EFS application and MAF administration, respectively. Summarized graphs showing the effects of MAF on (**B**) AUC and (**C**) amplitude of contractions. Significant difference (* *p* < 0.05 and *** *p* < 0.001) compared with control.

**Figure 5:**
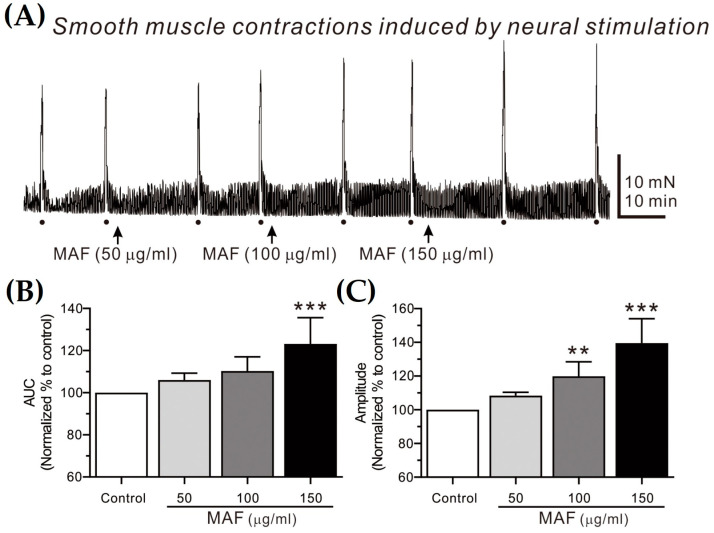
Effects of MAF on EFS-induced contractions of smooth muscle strips in human sigmoid colon in situ. (**A**) Representative raw traces showing that MAF increased electrically evoked contractions by neural stimulation in a dose-dependent manner (50–150 μg/mL). Dots and arrows indicate the points of EFS application and MAF administration, respectively. Summarized graphs showing the effects of MAF on (**B**) AUC and (**C**) amplitude of contractions. Significant difference (** *p* < 0.01 and *** *p* < 0.001) compared with control.

EFS-induced contractions of smooth muscle strips were recorded in situ in the human ileum (Figure 4) and sigmoid colon (Figure 5) and their AUC and amplitude for 10 s were analyzed (Table 2). The AUC and amplitude increased significantly by the administration of MAF in a dose-dependent manner for both ileal and colonic contractions. It is well-known that the EFS applied in the present study (i.e., 10 Hz, 10 s, 100 V, and 0.3 ms pulse) can evoke neural responses in GI smooth muscle strips, indicating that the administration of MAF significantly increased the neurogenic contractions in both the human ileal and colonic smooth muscles. These results suggest the potential for MAF to increase nerve-mediated mass contractions in the real human intestine. Therefore, we examined the effects of MAF on neurally mediated contractions occurring in human intestinal segments (see Section 2.7).

### 3.5. Effects of MAF on MMCs in Human Intestinal Segments

**Figure 6:**
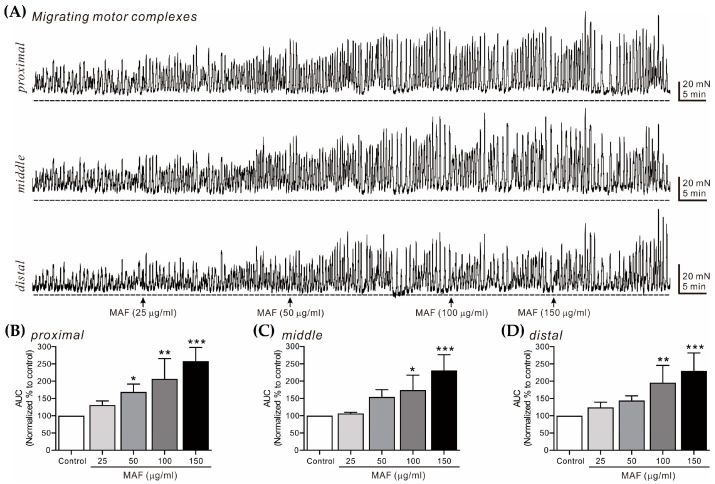
Effects of MAF on MMCs in human ileal segments in situ. (**A**) Representative raw traces showing that MAF increased MMCs in human ileum at the proximal, middle, and distal sites in a dose-dependent manner (25–150 μg/mL). Arrows indicate the points of MAF administration. Dotted lines indicate the baseline tension. (**B**–**D**) Summarized graphs showing the effects of MAF on AUC at three different sites of the ileal segment. Significant difference (* *p* < 0.05, ** *p* < 0.01, and *** *p* < 0.001) compared with control.

**Figure 7:**
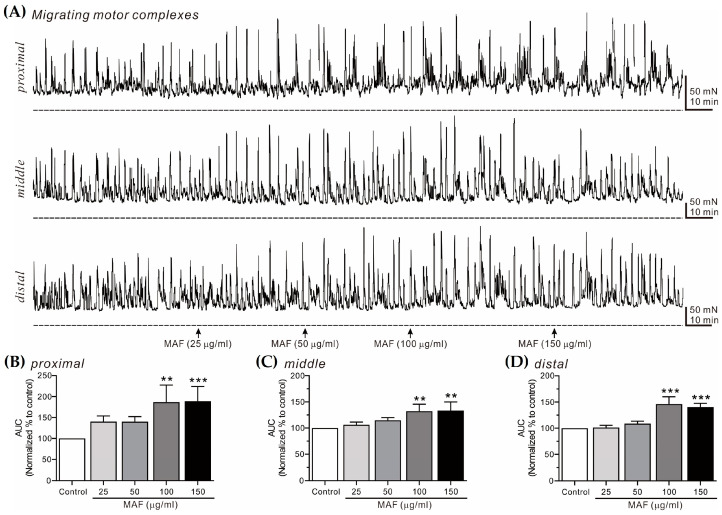
Effects of MAF on MMCs in human colonic segments in situ. (**A**) Representative raw traces showing that MAF increased MMCs in human sigmoid colon at the proximal, middle, and distal sites in a dose-dependent manner (25–150 μg/mL). Arrows indicate the points of MAF administration. Dotted lines indicate the baseline tension. (**B**–**D**) Summarized graphs showing the effects of MAF on AUC at three different sites of the colonic segment. Significant difference (** *p* < 0.01 and *** *p* < 0.001) compared with control.

MMCs are cyclic propulsive contractions observed in the GI tract and mediated by neural activities [37,38]. These contractions are also referred to as “propagating contractile complexes” or “peristalsis” [39]. Mass contractions, such as MMCs, move luminal contents distally, which is a very important contractile pattern in the digestive and defecating functions of the GI tract. In this study, MMCs from bowel segments were recorded in situ at 3 different sites (i.e., proximal, middle, and distal sites, 2 cm apart) in the human ileum (Figure 6) and sigmoid colon (Figure 7) and their AUC for 10 min was analyzed (Table 3). The AUC for both the ileal and colonic MMCs at all three different sites increased significantly by the administration of MAF in a dose-dependent manner, which was slightly more evident in the ileum than in the sigmoid colon at all three different sites. Interestingly, unlike the pattern of the ileal myogenic contractions (Figure 2A,C), the amplitude of the ileal MMCs increased apparently after the MAF administration (Figure 6A), implying that the MAF-induced increase in the ITR might be primarily related with the ileal MMCs rather than the ileal myogenic contractions.

## 4. Conclusions

In summary, the results in this study demonstrate that MAF significantly accelerated the in vivo ITR in mice. Moreover, the potency of MAF, in terms of the ITR, was significantly higher than that of cisapride or metoclopramide, suggesting that MAF has potential as a potent alternative to cisapride and metoclopramide for the treatment of GI motility disorders. This is particularly noteworthy because cisapride, the best-selling prokinetic agent in the late 1900s, was withdrawn from the market due to its cardiovascular side effects. For similar reasons, the use of metoclopramide in functional GI tract disorders was restricted in 2014. The effects of MAF on myogenic and neurogenic smooth muscle contractions in the human intestine were also investigated by measuring the spontaneous contractions of smooth muscle strips, smooth muscle contractions induced by neural stimulation, and MMCs from intestinal segments in the human ileum and sigmoid colon in situ. Both the myogenic and neurogenic contractions were increased by MAF to enhance the ileal and colonic motility in the human intestine. Taken together, these results indicate that MAF enhanced intestinal motility by increasing both myogenic and neurogenic contractions, thereby accelerating the ITR. These findings suggest that MAF could be a potential therapeutic agent for constipation or other diseases related to GI motility disorders. Further studies will be needed in regard to isolating the active component(s) from MAF, elucidating the contractile mechanisms, and evaluating the potential as an effective prokinetic agent for clinical application in human patients.

## Figures and Tables

**Table 1:** Relative values of AUC, amplitude, and frequency (% of control) for spontaneous contractions after treatment with each dose of MAF in human ileal and colonic smooth muscle strips in situ.

Segment	Parameter (*n*)	Dose of MAF (μg/mL)			
		25	50	100	150
Ileum	AUC (8)	132.00 ± 6.77	190.88 ± 13.46 **	255.57 ± 20.04 ***	293.00 ± 34.65 ***
	Amplitude (9)	115.11 ± 6.86	132.10 ± 13.17	128.89 ± 10.67	130.11 ± 11.27
	Frequency (6)	143.67 ± 20.95	243.00 ± 39.94 *	333.60 ± 30.17 ***	425.00 ± 55.38 ***
Sigmoid colon	AUC (7)	131.29 ± 9.55	181.29 ± 15.53 **	227.86 ± 21.08 ***	266.43 ± 27.88 ***
	Amplitude (6)	133.50 ± 12.49	176.00 ± 13.01 **	191.17 ± 15.96 ***	229.83 ± 25.33 ***
	Frequency (6)	158.80 ± 18.69	185.80 ± 21.98 *	214.00 ± 27.52 **	224.80 ± 31.98 **

Data are expressed as the mean ± SEM (% of each control). * *p* < 0.05, ** *p* < 0.01, and *** *p* < 0.001 vs. each control.

**Table 2:** Relative values of AUC and amplitude (% of control) for EFS-induced contractions after treatment with each dose of MAF in human ileal and colonic smooth muscle strips in situ.

Segment	Parameter (*n*)	Dose of MAF (μg/mL)		
		50	100	150
Ileum	AUC (5)	121.40 ± 4.58 *	133.80 ± 5.95 ***	148.60 ± 8.76 ***
	Amplitude (5)	118.20 ± 3.97 *	129.80 ± 4.75 ***	143.00 ± 6.76 ***
Sigmoid colon	AUC (6)	106.00 ± 3.32	110.29 ± 2.56	123.14 ± 4.73 ***
	Amplitude (6)	108.33 ± 2.08	119.83 ± 3.54 **	139.50 ± 5.94 ***

Data are expressed as the mean ± SEM (% of each control). * *p* < 0.05, ** *p* < 0.01, and *** *p* < 0.001 vs. each control.

**Table 3:** Relative AUC values (% of control) for MMCs after treatment with each dose of MAF in human ileal and colonic segments in situ.

Segment	Site	Dose of MAF (μg/mL)			
		25	50	100	150
Ileum	Proximal	131.18 ± 11.66	168.83 ± 22.71 *	206.53 ± 26.27 **	258.07 ± 17.70 ***
	Middle	106.50 ± 3.48	154.00 ± 21.19	174.00 ± 21.74 *	230.75 ± 22.85 ***
	Distal	124.00 ± 15.40	144.00 ± 13.68	195.50 ± 25.05 **	229.75 ± 25.99 ***
Sigmoid colon	Proximal	140.40 ± 13.43	140.00 ± 12.28	186.75 ± 20.40 **	189.25 ± 17.39 ***
	Middle	106.20 ± 5.18	114.80 ± 5.06	132.00 ± 6.10 **	133.20 ± 7.44 **
	Distal	101.25 ± 4.39	108.75 ± 4.61	146.00 ± 7.08 ***	140.25 ± 3.71 ***

Data are expressed as the mean ± SEM (% of each control, *n* = 5 for each value). * *p* < 0.05, ** *p* < 0.01, and *** *p* < 0.001 vs. each control.

## Data Availability

The data presented in this study are available from the corresponding author upon request.

## Supplementary Materials

The following supporting information can be downloaded at: https://www.mdpi.com/article/10.3390/nu15081889/s1, Figure S1: An experimental setup for recording tension from a colonic segment.
