# Supplementary figures and images for: Prokinetic Activity of Mulberry Fruit, Morus alba L
Source: Nutrients. 2023 Apr 14;15(8):1889. doi: 10.3390/nu15081889 (PMC10143206; doi:10.3390/nu15081889)

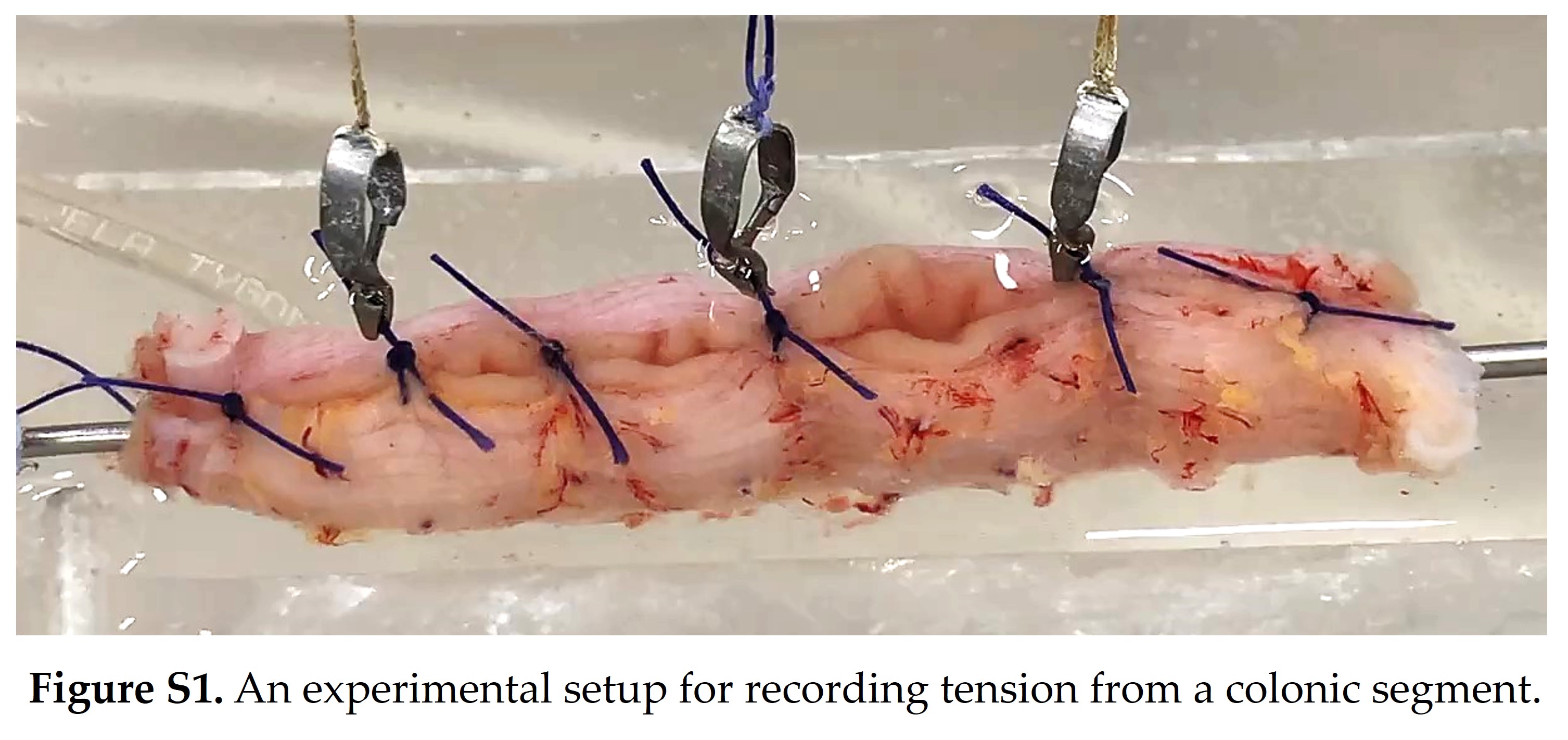

Supplement: Supplementary file 1 [file nutrients-15-01889-s001.zip › nutrients-2316460-supplementary.jpg]
